# Revisiting post-ICU admission fluid balance across pediatric sepsis mortality risk strata: A secondary analyses from a prospective observational cohort study

**DOI:** 10.21203/rs.3.rs-3117188/v1

**Published:** 2023-06-28

**Authors:** Mihir R. Atreya, Natalie Z. Cvijanovich, Julie C. Fitzgerald, Scott L. Weiss, Michael T. Bigham, Parag N. Jain, Kamal Abulebda, Riad Lutfi, Jeffrey Nowak, Neal J. Thomas, Torrey Baines, Michael Quasney, Bereketeab Haileselassie, Rashmi Sahay, Bin Zhang, Matthew Alder, Natalja Stanski, Stuart Goldstein

**Affiliations:** Cincinnati Children’s Hospital Medical Center; UCSF Benioff Children’s Hospital Oakland, Oakland, CA 94609, USA.; Children’s Hospital of Philadelphia, Philadelphia, PA 19104, USA.; 5. Nemours Children’s Hospital, Wilmington, DE, 19803, USA.; Akron Children’s Hospital, Akron, OH 44308, USA.; Texas Children’s Hospital and Baylor College of Medicine, Houston, TX 77030, USA.; Riley Hospital for Children, Indianapolis, IN 46202, USA.; Riley Hospital for Children, Indianapolis, IN 46202, USA.; Children’s Hospital and Clinics of Minnesota, Minneapolis, MN 55404, USA.; Penn State Hershey Children’s Hospital, Hershey, PA 17033, USA.; University of Florida Health Shands Children’s Hospital, Gainesville, FL 32610, USA.; CS Mott Children’s Hospital at the University of Michigan, Ann Arbor, MI 48109, USA.; Lucile Packard Children’s Hospital Stanford, Palo Alto, CA 94304, USA.; Cincinnati Children’s Hospital Medical Center; Cincinnati Children’s Hospital Medical Center; Cincinnati Children’s Hospital Medical Center; Cincinnati Children’s Hospital Medical Center; Cincinnati Children’s Hospital Medical Center

**Keywords:** Critical illness, Sepsis, Septic Shock, Acute Kidney Injury, Post-ICU Admission Fluid Balance, Positive Fluid Balance, Fluid Overload, Biomarkers

## Abstract

**Introduction:**

Post-ICU admission cumulative positive fluid balance (PFB) is associated with increased mortality among critically ill patients. We sought to test whether this risk varied across biomarker-based risk strata upon adjusting for illness severity, presence of severe acute kidney injury (AKI), and use of renal replacement therapy (CRRT) in pediatric septic shock.

**Design:**

Ongoing multi-center prospective observational cohort.

**Setting:**

Thirteen pediatric ICUs in the United States (2003–2023).

**Patients:**

Six hundred and eighty-one children with septic shock.

**Interventions:**

None.

**Measurements and Main Results:**

Cumulative percent positive fluid balance between day 1–7 (Day 1–7%PFB) was determined. Primary outcome of interest was complicated course defined as death or persistence of ≥ 2 organ dysfunctions by day 7. PERSEVERE-II biomarkers were used to assign mortality probability and categorize patients into high (n = 91), intermediate (n = 134), and low (n = 456) mortality risk strata. Cox proportional hazard regression models with adjustment for PERSEVERE-II mortality probability, presence of sepsis associated acute kidney injury (SA-AKI) on Day 3, and any use of CRRT, demonstrated that time-dependent variable Day 1–7%PFB was independently associated with increased hazard of complicated course in the cohort. Risk stratified analyses revealed that each 10% increase in Day 1–7%PFB was independently associated with increased hazard of complicated course among patients with high mortality risk strata (adj HR of 1.24 (95%CI: 1.08–1.42), p = 0.002), but not among those categorized as intermediate- or low- mortality risk.

**Conclusions:**

Our data demonstrate the independent influence of cumulative %PFB on the risk of complicated course. Contrary to our previous report, this risk was largely driven by patients categorized as having a high-mortality risk based on PERSEVERE-II biomarkers. Further research is necessary to determine whether this subset of patients may benefit from targeted deployment of restrictive fluid management or early initiation of de-escalation therapies upon resolution of shock.

## Introduction

Sepsis is associated with high morbidity and mortality among children and adults.(1) Driven by a dysregulated host immune response and consequent endothelial activation, sepsis is characterized by microvascular capillary leak.(2) A large increase in systemic vascular capacitance necessitates fluid resuscitation to ensure adequate preload and systemic oxygen delivery in the initial phase of illness. However, fluid shifts over the course of disease result in extravascular fluid accumulation, consequent hypoxemia, and organ dysfunction(s).(3)

Persistence of a positive fluid balance (PFB) has been independently associated with poor clinical outcomes among patients with sepsis (4–9). Complicating matters further, patients may develop sepsis associated acute kidney injury (SA-AKI) and oliguria. A subset of patients may require continuous renal replacement therapy (CRRT). Both presence of SA-AKI and CRRT use may serve to confound the association between cumulative PFB and clinical outcomes. Recent research has emphasized conservative fluid management, de-escalation, and deresuscitation where appropriate to negate the detrimental effects of positive fluid balance among critically ill patients.(10) It is suggested that such approaches may improve liberation of patients from intensive care units (ICU). A key limitation is that few studies have explicitly considered biological heterogeneity among critically ill patients. It is likely that subsets of patients may benefit from tailored fluid management approaches.(11–13)

Our group has previously utilized Pediatric Sepsis Biomarker Risk Model (PERSEVERE) — a prospectively validated prognostic enrichment tool(14) reflective of the host inflammatory response—to the test the association between post-ICU admission cumulative PFB across mortality risk strata in pediatric septic shock patients.(15) We reported that only in patients with low-mortality risk was cumulative PFB associated with an increased odds of complicated course- a composite of death and multiple organ dysfunctions. Limitations of our prior study included: 1) reporting of univariate analyses that did not account for potential confounders, 2) the lack of consideration of the competing influence of early deaths on ICU length of stay, and consequently on the cumulative PFB among the most critically ill patients, and 3) the relatively small number of patients belonged to high- and intermediate- PERSEVERE mortality risk strata.

In this study, we sought to test the independent influence of post-ICU admission PFB on the risk of complicated course in a large cohort of children with septic shock. We further conducted risk-stratified analyses to test differences in this association across PERSEVERE-II mortality risk strata.

## Methods

### Study design and patient selection:

The study protocol was approved by Institutional Review Boards (IRBs) of participating institutions (Cincinnati Children’s Hospital IR, Genomic Analysis of Pediatric Systemic Inflammatory Syndrome, IRB ID: 2008 − 0558, Continuous Review) as well as all participating institutions. Informed consent was obtained from parent or guardian of patients. All procedures performed in studies involving human participants were in accordance with the ethical standards of the institutional review boards of participating institutions and with the 1964 Helsinki declaration and its later amendments or comparable ethical standards.

Our ongoing multi-institutional prospective observational cohort study of pediatric septic shock has been extensively detailed previously. (14, 16, 17) Inclusion criteria for this study was meeting pediatric-specific consensus criteria for septic shock.(18) Those with (1) missing admission height to estimate baseline serum creatinine or weight to estimate percent PFB per kilogram body weight, (2) those with missing fluid balance details on day 1, and those without (3) PERSEVERE-II biomarker data were excluded. Baseline illness severity was based on PRISM-III score.(19) The percent positive fluid balance was measured by dividing the net fluid balance (intake minus output) measured in liters on any given day by the admission weight in kilograms and multiplying it by a factor of 100. The primary exposure variable of interest was the cumulative percent positive fluid balance between days 1 and 7 (Day 1–7%PFB). The primary outcome of interest was complicated course –defined as death within 7 days or the presence of 2 or more organ dysfunctions on day 7 of septic shock.(17) Severe SA-AKI was defined as per Kidney Disease Improving Global Outcomes (KDIGO) stage 2 AKI or higher, which corresponds to a two-fold or greater increase in serum creatinine (SCr) relative to baseline.(20) Baseline SCr values were unknown for all patients in the cohort, and thus were imputed using their calculated body surface area (m2) and an eGFR of 120 ml/min per 1.73 m2, as validated in the literature.^19, 20^ Presence of persistent SA-AKI on day 3 (D3 SA-AKI) and use of any CRRT between days 1–7 were considered as co-variates.

#### PERSEVERE-II based Risk Stratification

PERSEVERE-II mortality probability and risk strata were determined, according to published methods.(14, 21) Briefly, Interleukin-8 (IL-8), Heat shock protein 70 kDA (HSP70), C-C Chemokine ligand 3 (CCL3), C-C Chemokine ligand 4 (CCL4), Granzyme B (GZMB), Interleukin-1 α (IL-1a), Matrix metallopeptidase 8 (MMP8) were previously measured in day 1 septic shock serum. Classification And Regression Tree (CART) analyses were used to derive a mortality probability risk score (0.000–0.999) using R. Patients were Classified as low risk (mortality probability score ≤ 0.019), intermediate risk (mortality probability score > 0.019 to ≤ 0.300) or high risk (mortality probability score > 0.300).

#### Statistical analyses

Statistically analyses were conducted using R software. Demographic and clinical data were summarized with numbers with percentages or median with interquartile range. Differences between groups were determined by χ^2^ squared test for categorical variables and by Kruskal Wallis test for continuous variables with post-hoc Dunn’s test for multiple comparison testing between risk strata. Univariate and multi-variable Cox proportional regression were used to estimate hazard of complicated course in the cohort and across PERSEVERE-II risk strata. Models were adjusted for D3 SA-AKI, use of CRRT, with Day 1–7% PFB being considered a time-dependent variable. Day of study enrollment was considered as the starting time (Time 0). Survivors who were transferred out of the ICU before day 7 and those who remained in the ICU on day 7 were right censored. A p-value of 0.05 was used to test statistical significance throughout the study.

## Results

**Figure 1:**
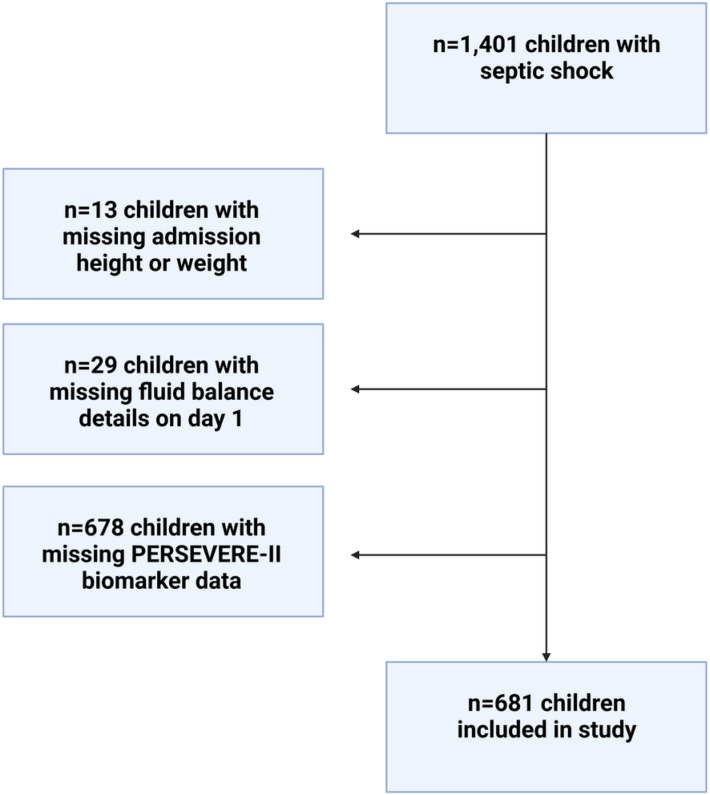
Flow diagram showing patients included in the study.

A total of 681 children met inclusion criteria of this study. Figure 1 shows flow diagram detailing patients who were included in the study; 13 children with missing admission height or weight, 29 children with missing fluid balance on day 1, and 678 children without PERSEVERE-II biomarker data were excluded from analyses. Demographic and clinical variables based on presence of complicated course are presented in Table 1. Patients with complicated course had higher 28-day mortality, hospital length of stay, presence of D3 SA-AKI, and use of any CRRT. Percent PFB was higher between days 1 and 4 among those with complicated course. Net daily even or negative fluid balance was achieved later among patients with complicated course than to those without. Finally, among patients who received CRRT, duration of CRRT use was longer among those with complicated course than those without.

Differences in demographic and clinical characteristics according to PERSEVERE-II risk strata are shown in Table 2; 91 patients were deemed high-risk, 134 patients had intermediate-risk, and 456 patients had low-mortality risk. Patients categorized as high- and intermediate- risk categories had higher illness severity based on both PRISM-III score and PERSEVERE-II mortality probability. Thes patients also had worse clinical outcomes including the presence of D3 SA-AKI and use of CRRT with graded responses across risk strata. Relative to those classified as low-mortality risk, fluid balance between day 1–3 and cumulatively over the first 7 days were more positive among patients with high- and intermediate risk groups, with no differences between the latter groups noted. Table 3 shows the results of univariate and multivariate Cox proportional hazard regression for complicated course in the entire cohort. After adjusting for PERSEVERE-II mortality probability, D3 SA-AKI, and use of CRRT, every 10% percent increase in Day 1–7%PFB was associated with an increase in hazard of complicated course [adj. HR 1.10 (95% CI: 1.01–1.20), p = 0.042]. Importantly, PERSEVERE-II mortality probability and presence of D3 SA-AKI, but not use of CRRT, were independently associated with an increased hazard of complicated course.

**Figure 2:**
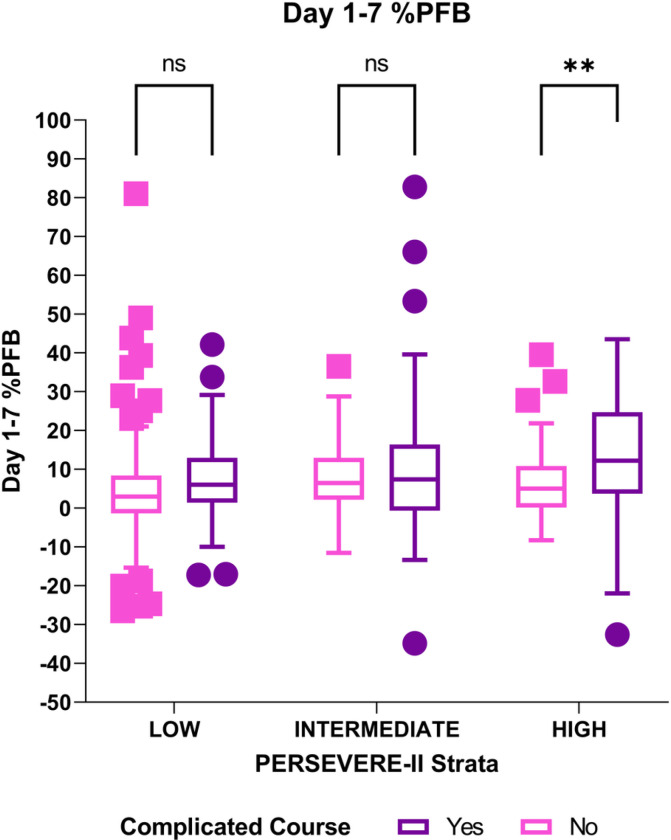
Day 1–7 cumulative percent fluid overload by presence of complicated course across PERSEVERE-II risk strata. Patients with complicated course had higher Day 1–7 %PFB than those without only among patients categorized as high PERSEVERE-II mortality risk.

Figure 2 shows Day 1–7%PFB according to occurrence of complicated course and PERSEVERE-II mortality strata. Only patients belonging to the high-mortality risk group with complicated course had higher Day 1–7%PFB. Table 4 shows the results of univariate and multivariate Cox proportional hazard for complicated course for each of the three risk-strata. After adjusting for the potentially confounding influence of D3 SA-AKI and use of CRRT, every 10% increase in Day 1–7% PFB was associated with an increased hazard [adj HR of 1.24 (95%CI: 1.08–1.43), p = 0.003] of complicated course among patients belonging to high PERSEVERE-II mortality risk, but not among those categorized as intermediate (p = 0.85) or low (p = 0.39) mortality risk. The presence of D3 SA-AKI was independently associated with increased hazard of complicated course among patients with high- and low-mortality risk but not among patients with intermediate mortality risk. Use of CRRT did not influence risk of complicated course in any of the mortality risk groups.

## Discussion

We provide evidence that cumulative positive fluid balance is independently associated with increased hazard of death and multiple organ dysfunctions among pediatric septic shock patients. Further, risk-stratified analyses based on PERSEVERE-II biomarkers demonstrated that this increased risk is attributable primarily to patients categorized as having a high-risk.

Numerous studies among adults have sought to address the link between positive fluid balance and clinical outcomes in the previous decade. Data from a recent meta-analyses of 31 observational studies and 3 randomized trials showed an independent association between positive cumulative fluid balance and mortality.(22) However, there was substantial variation in adjustment for confounding factors with most accounting for illness severity and some adjusting for use and timing of CRRT. Few studies have adjusted for the concomitant presence of AKI. (23) Recent results from the assessment of worldwide acute kidney injury renal angina and epidemiology (AWARE) study from over 5,000 critically ill children, demonstrate that mild-to-moderate fluid overload as early at the end of the ICU day 1 was associated with adverse outcomes including higher mortality and fewer ICU and ventilator free days.(9) Our current data from a prospective observational study from multiple-pediatric centers are corroborative.

Few studies have considered heterogeneous responses across critical illness subclasses or risk-strata with regard to cumulative fluid balance and mortality outcomes. Through latent profile analyses of electronic health record data, Zhang and colleagues showed that sepsis “profile 3”, characterized by shock and multiple organ dysfunctions, demonstrated a survival benefit with a higher cumulative fluid balance in the first 48 hours. In contrast, “profile 4” demonstrated an increased odds of death with a more positive fluid balance.(11) Wang et al. studied fluid balance trajectories among critically ill patients a significant proportion of whom had sepsis. They demonstrated that relative to patients with a low fluid balance, those with a high fluid balance demonstrated an increased odds of mortality. In contrast, patients with a “decreasing” fluid balance trajectory demonstrated a survival benefit.(24) Our data contradict those previously published by our group, where we reported worse outcomes with high positive fluid balance among those categorized as having a low biomarker-based mortality risk strata alone.(15) There are several important contributors to the differences between direction of association including 1) larger sample size to allow for detection of statistically significant differences among patients with high-mortality risk, 2) use of multivariable models that account for D3 SA-AKI and CRRT, and 3) use of Cox proportional model to account for the competing influence of death on ICU survival and therefore on cumulative fluid balance. An advantage of our data is that biomarkers were collected on day 1 of illness with subsequent assessment of cumulative fluid balance. Our data show that patients with the highest burden of systemic inflammation as indicated by PERSEVERE-II biomarkers on day 1 of illness, were more likely to have had a positive fluid balance in the post-resuscitative and stabilization phases of septic shock and the worst outcomes. Given the observational nature of our study, we cannot make any inferences on whether PFB causally mediates the influences between biomarker-based risk strata assignment and worse clinical outcomes.

A recent randomized trial of restrictive vs. standard fluid management in patients with septic shock demonstrated no mortality benefit.(25) It is conceivable, however, that given the heterogeneous nature of sepsis, a subset of patients with a higher degree of systemic inflammation and endothelial activation may in fact demonstrate a differential response to restrictive fluid management. Accordingly, our data among patients with high-mortality risk based on PERSEVERE-II biomarkers warrant further investigation to determine whether these patients may selectively benefit from restrictive fluid management with early initiation of vasoactive support or alternatively, such patients may benefit from early de-escalation or de-resuscitation upon resolution of shock.

Our study has several limitations: 1) data on resuscitative fluids received outside of the pediatric ICU were not available and thus not included, 2) fluid choice (crystalloid vs colloid, isotonic vs non-isotonic) were not documented and could not be controlled for, 3) CRRT prescription data were not available, 4) given the relatively limited number of patients on CRRT, we did not adjust for duration and timing of CRRT in multivariate regression models. Each of these potential confounding variables have been shown in other studies to potentially influence the association between post-ICU admission positive fluid balance and clinical outcomes.

## Conclusions

Cumulative positive fluid balance is independently associated with worse clinical outcomes among children with septic shock after adjusting for illness severity, presence of severe AKI, and use of CRRT. Risk-stratified analyses demonstrated that patients with a high mortality risk, based on PERSEVERE-II biomarkers, primarily contributed to this association. Our data warrant further observation to determine whether this subset of patients may benefit from targeted deployment of restricted fluid management coupled with early initiation of vasoactive support or early de-escalation approaches upon clinical resolution of shock.

## Figures and Tables

**Table 1:** Demographics and clinical outcomes of cohort based on presence of complicated course.

Complicated course	Yes (N = 216)	No (N = 465)	P value
Age (Years)	6.3 (1.9, 12.7)	3.9 (1.0, 8.9)	< 0.001
Weight	16.4 (8.6, 25.8)	20.9 (12.3, 37.1)	< 0.001
Sex, Female (%)	100 (46.3%)	230 (49.4%)	0.442
Race (Self-identified)			
White/Caucasian	156 (72.2%)	349 (75.1%)	0.467
Black/African American	23 (10.7%)	53 (11.4%)	
Other	37 (17.1%)	63 (13.5%)	
Ethnicity			
Hispanic or Latino	28 (12.9%)	63 (13.6%)	0.933
PRISM-III	13 (8, 22)	9 (5, 13)	< 0.001
PERSEVERE-II mortality risk	0.167 (0.148–0.186)	0.074 (0.062–0.087)	< 0.001
Day 1 VIS	23 (9, 50)	10 (3, 30)	< 0.001
28-day mortality	70 (32.4%)	4 (0.9%)	< 0.001
PICU Free days	15 (6, 21)	24 (20, 26)	< 0.001
Hospital LOS	21 (9, 30)	12 (7, 21)	< 0.001
D3 SA-AKI	139 (62.6%)	83 (17.8%)	< 0.001
Any CRRT	53 (24.5%)	19 (4.1%)	< 0.001
Day 1%FB	5.6 (2.6, 9.6)	3.2 (0.6, 6.4)	< 0.001
FB: Fluid balance			
SA-AKI: Sepsis associated acute kidney injury			
CRRT: Continuous renal replacement therapy.			
Day 2%FB	3.4 (0.7, 7.0)	1.5 (−0.7, 3.9)	< 0.001
Day 3%FB	0.2 (−1.5, 3.7)	0.1 (−2.1, 2.1)	0.038
Day 4%FB	0.3 (−2.8, 3.3)	−0.4 (−3.1, 1.2)	0.007
Day 5%FB	−0.1 (−2.9, 1.8)	−0.4 (−2.4, 1.6)	0.782
Day 6%FB	−0.6 (−3.3, 1.2)	0.2 (−1.7, 2.0)	0.001
Day 7%FB	−0.2 (−2.3, 2.1)	0.4 (−1.4, 2.2)	0.072
Day 1–7%PFB	7.5 (1.5, 15.3)	3.6 (−0.8, 9.1)	< 0.001
Time to achieve daily even or negative fluid balance (Days)	3 (2, 3)	2 (1, 2)	< 0.001
Duration of CRRT (Hours)	31 (25–36)	4 (1–8)	< 0.001
FB: Fluid balance			
SA-AKI: Sepsis associated acute kidney injury			
CRRT: Continuous renal replacement therapy.			

**Table 2:** Demographics and clinical outcomes of cohort by PERSEVERE-II risk strata.

	High risk (n=91)	intermediate risk (n = 134)	Low risk (n = 456)	P value
Age (Years)	4.4 (1.5, 8.2)	5.4 (1.5, 9.6)	5.6 (1.6, 10.8)	0.246
Weight	17.1 (10.5, 24.8)	18.5 (10.3, 33.0)	20.0 (11.3, 35.7)	0.414
Sex, Female (%)	48 (52.7%)	59 (44.1%)	223 (48.9%)	0.415
Race (Self-identified)				
White/Caucasian	69 (75.8%)	100 (74.6%)	336 (73.7%)	0.866
Black/African American	8 (8.8%)	13 (9.7%)	55 (12.1%)	
Other	14 (15.4%)	21 (15.7%)	65 (14.3%)	
Ethnicity				
Hispanic or Latino	8 (8.8%)	18 (13.4%)	65 (14.3%)	0.715
PRISM-III	16 (9, 26) **	13 (8, 18) *	9 (5, 13)	< 0.001
PERSEVERE-II mortality risk	0.407 (0.07) **	0.186 (0.01) *	0.02 (0.07)	< 0.001
Day 1 VIS	20 (4, 55) *	23 (10, 54) *	10 (3, 30)	< 0.001
Complicated Course	51 (56.1%) *	58 (43.2%) *	107 (23.5%)	< 0.001
7-day mortality	21 (23.1%) *	18 (13.4%) *	9 (1.9%)	< 0.001
28-day mortality	30 (32.9%)	23 (17.2%)	21 (4.6%)	< 0.001
PICU Free days	23 (16, 26)	21 (13, 25)	23 (16, 26)	0.308
Hospital LOS	13 (5, 27)	16 (8, 27)	13 (7, 25)	0.476
D3 SA-AKI	46 (50.5%) **	53 (39.6%) *	123 (26.8%)	< 0.001
Any RRT	19 (20.8%) **	21 (15.7%) *	32 (7.0%)	< 0.001
Day 1%FB	6.8 (5.7, 7.9) *	6.3 (5.4, 7.2) *	4.7 (3.2, 4.2)	< 0.001
Day 2%FB	3.8 (2.8, 4.8) *	4.1 (3.3, 4.9) *	4.4 (1.3, 2.2)	< 0.001
Day 3%FB	1.4 (0.4, 2.4) *	0.5 (−0.3, 1.3)	0.0 (−0.4, 0.5)	0.032
Day 4%FB	−0.3 (−1.3, 0.6)	−0.8 (−1.6, 0.0)	−0.1 (−0.5, 0.3)	0.315
Day 5%FB	−0.2 (−0.9, 0.5)	−0.3 (−0.8, 0.3)	−0.4 (−0.7, −0.1)	0.764
Day 6%FB	−0.5 (−1.1, 0.2)	−0.1 (−0.5, 0.5)	−0.2 (−0.5, 0.1)	0.609
Day 7%FB	0.5 (−0.5, 1.4)	0.1 (−0.7, 0.9)	−0.1 (−0.5, 0.3)	0.571
Day 1–7%PFB	11.5 (8.7, 14.3) *	9.9 (7.6, 12.2) *	4.6 (3.4, 5.9)	< 0.001
Days to achieve daily even or negative fluid balance	3 (2, 3) *	3 (2, 3) *	2 (1, 2)	< 0.001
Duration of CRRT (hours)	29 (21–37) *	16 (10–23)	8 (5–12)	< 0.001

**Table 3:** Cox Proportional Hazard Model for Complicated Course for the entire cohort.

	HR (95% CI)	P value
**Univariate models:**		
Age	1.00 (0.98–1.02)	0.66
P-II Mortality Probability*	1.26 (1.17–1.37)	<0.001
RRT	1.82 (1.34–2.49)	<0.001
D3 SA-AKI	2.68 (2.03–3.54)	<0.001
Day 1–7% PFB*	1.10 (1.01–1.20)	0.002
**Multivariate model:**		
P-II Mortality Probability	1.15 (1.05–1.26)	0.003
RRT	1.01 (0.70–1.46)	0.961
D3 SA-AKI	2.27 (1.67–3.09)	<0.001
Day 1–7% PFB*	1.10 (1.01–1.20)	0.042
Day 1–7%PFB was multiplied by factor of 10 for models.		

**Table 4:** Cox Proportional Hazard Models for Complicated Course by PERSEVERE-II Mortality Risk Strata.

PERSEVERE-II Risk Strata	HIGH RISK		INTERMEDIATE RISK		LOW RISK	
	HR (95% CI)	P value	HR (95% CI)	P value	HR (95% CI)	P value
**Univariate models:**						
Age	1.00 (0.96–1.03)	0.787	1.01 (0.97–1.05)	0.644	0.99 (0.96–1.02)	0.427
Any RRT	1.04 (0.57–1.88)	0.898	1.65 (0.94–2.90)	0.084	2.00 (1.23–3.25)	0.005
D3 SA-AKI	2.18 (1.14–4.19)	0.019	1.84 (1.10–3.10)	0.021	2.94 (1.99–4.35)	< 0.001
Day 1–7% PFB*	1.24 (1.08–1.42)	0.002	1.04 (0.94–1.16)	0.453	1.09 (0.94–1.26)	0.282
**Multivariate model:**						
Any RRT	0.63 (0.32–1.25)	0.187	1.58 (0.85–2.94)	0.153	1.28 (0.77–2.16)	0.345
D3 SA-AKI	2.37 (1.16–4.87)	0.019	1.45 (0.83–2.55)	0.196	2.67 (1.77–4.02)	< 0.001
Day 1–7% PFB*	1.24 (1.08–1.43)	0.003	1.01 (0.91–1.12)	0.856	1.07 (0.91–1.26)	0.396
Day 1–7% PFB was multiplied by a factor of 10 for models.						

## Key Points

### Question:

We sought to test whether the association between cumulative positive fluid balance and complicated PICU course varied across biomarker-based risk strata among patients with pediatric septic shock.

### Finding:

After adjusting for illness severity, sepsis associated acute kidney injury, and use of continuous renal replacement therapy, cumulative positive fluid balance after PICU admission was independently associated with increased hazard of complicated course. This association was attributable primarily to patients categorized as having a high-mortality risk based on PERSEVERE-II biomarkers.

### Meaning:

Future research is necessary to determine whether patients deemed high-mortality risk benefit from restrictive fluid management or alternatively targeted de-escalation strategies.
